# Supplementary material for: HP1α targets the chromosomal passenger complex for activation at heterochromatin before mitotic entry
Source: EMBO J. 2018 Feb 21;37(6):e97677. doi: 10.15252/embj.201797677 (PMC5852645; doi:10.15252/embj.201797677)
Supplement: Supplementary file 1 — Expanded View Figures PDF [file EMBJ-37-e97677-s001.pdf]

Expanded View Figures

Figure EV1. Localisation properties of the various tethering constructs.

A, B HeLa cells expressing the indicated HP1α fusion constructs, stained with Hoechst 33342 and immunostained for CENP-C after pre-extraction. Line scan showing the expressed HP1α construct and CENP-C (i); the expressed HP1α construct only (ii); CENP-C only (iii). Scale bars, 5 μm.

C Alignment of the CENP-B DNA-binding domain protein sequences from various species. Multiple sequence alignment was performed using Clustal Omega and edited with Aline for display. The following species were used for the alignment: *Homo sapiens*, *Mus musculus*, *Cricetulus griseus*, *Rattus norvegicus*, *Schizosaccharomyces pombe* (CENP-B homolog proteins 1 and 2), *Ornithorhynchus anatinus*, *Bos taurus*, *Cavia porcellus*, *Macaca mulatta* and *Pediculus humanus*.

D Structure of the CENP-B DNA-binding domain is shown as a ribbon diagram interacting with DNA (PDB code 1HLV). Mutated residues are shown in stick.

E HeLa cells expressing the indicated HP1α fusion constructs, stained with Hoechst 33342 and immunostained for CENP-C. Scale bar, 5 μm.

F Immunoblot of HeLa cell lysates transfected with the indicated HP1α fusion constructs. HP1α fusion constructs were detected with anti-GFP antibody and α-tubulin was used as a loading control.

© 2018 The Authors

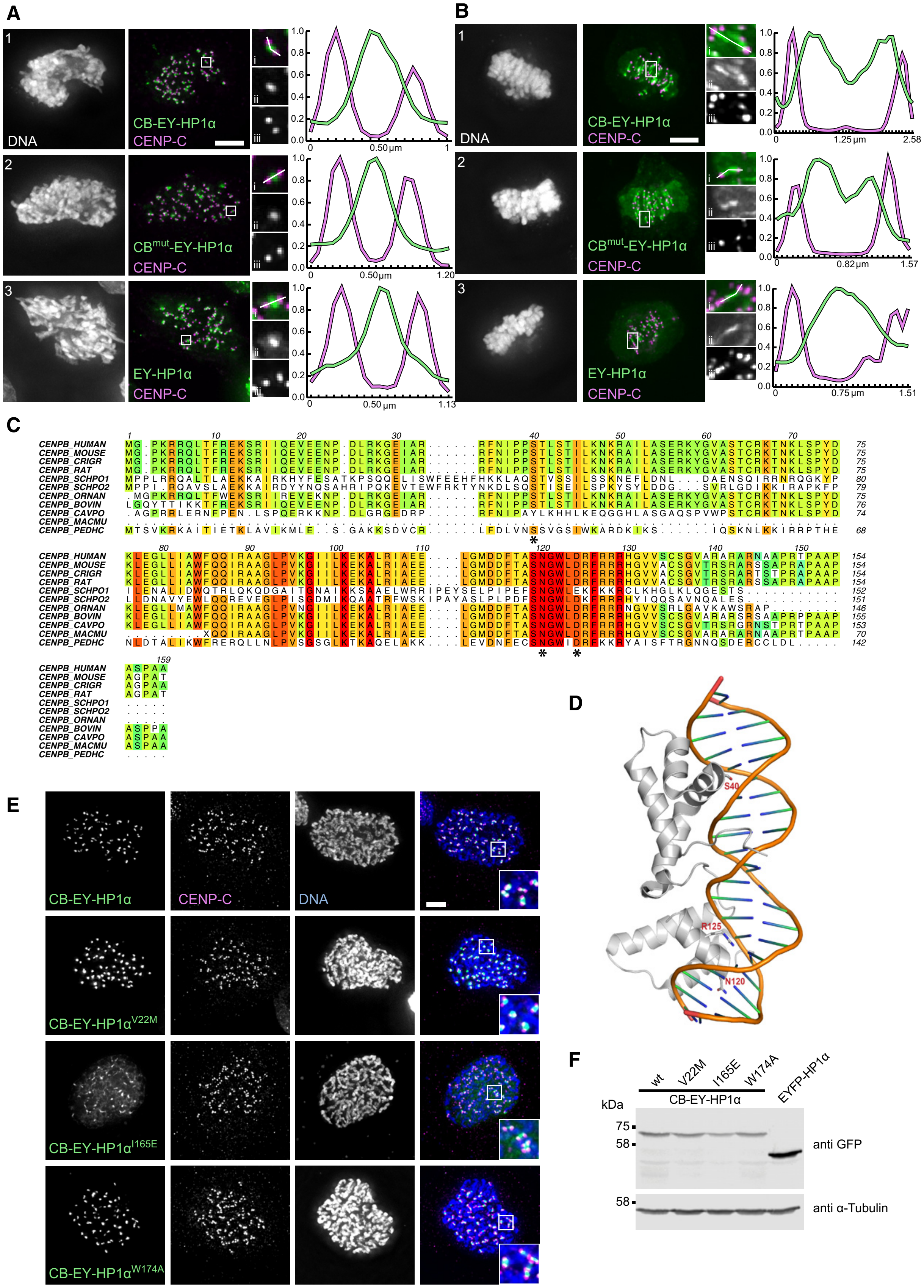

Figure EV1.

 © 2018 The Authors

Figure EV2. Constitutive retention of HP1-bound CPC results in H3S10 phosphorylation in interphase that is sensitive to low concentrations of ZM447439.

A, B HeLa cells expressing the wild-type HP1α (1) or mutated HP1α^{W174A} (2) tethering construct at the indicated stages, stained with Hoechst 33342 and immunostained for Aurora B and α-tubulin. The image brightness of the EYFP channel was scaled individually to optimise the clarity of the protein localisation, but in both examples, the CB-EY-HP1α^{W174A} was expressed at higher levels. Scale bars, 5 μm.

C, D HeLa cells expressing the wild-type HP1α tethering construct treated with either DMSO (1) or with 0.5 μM ZM447439 (2) for 60 min before fixation, stained with Hoechst 33342 and immunostained for H3S10ph and α-tubulin (C) or Aurora B and H3S10ph (D). Scale bars, 10 μm.

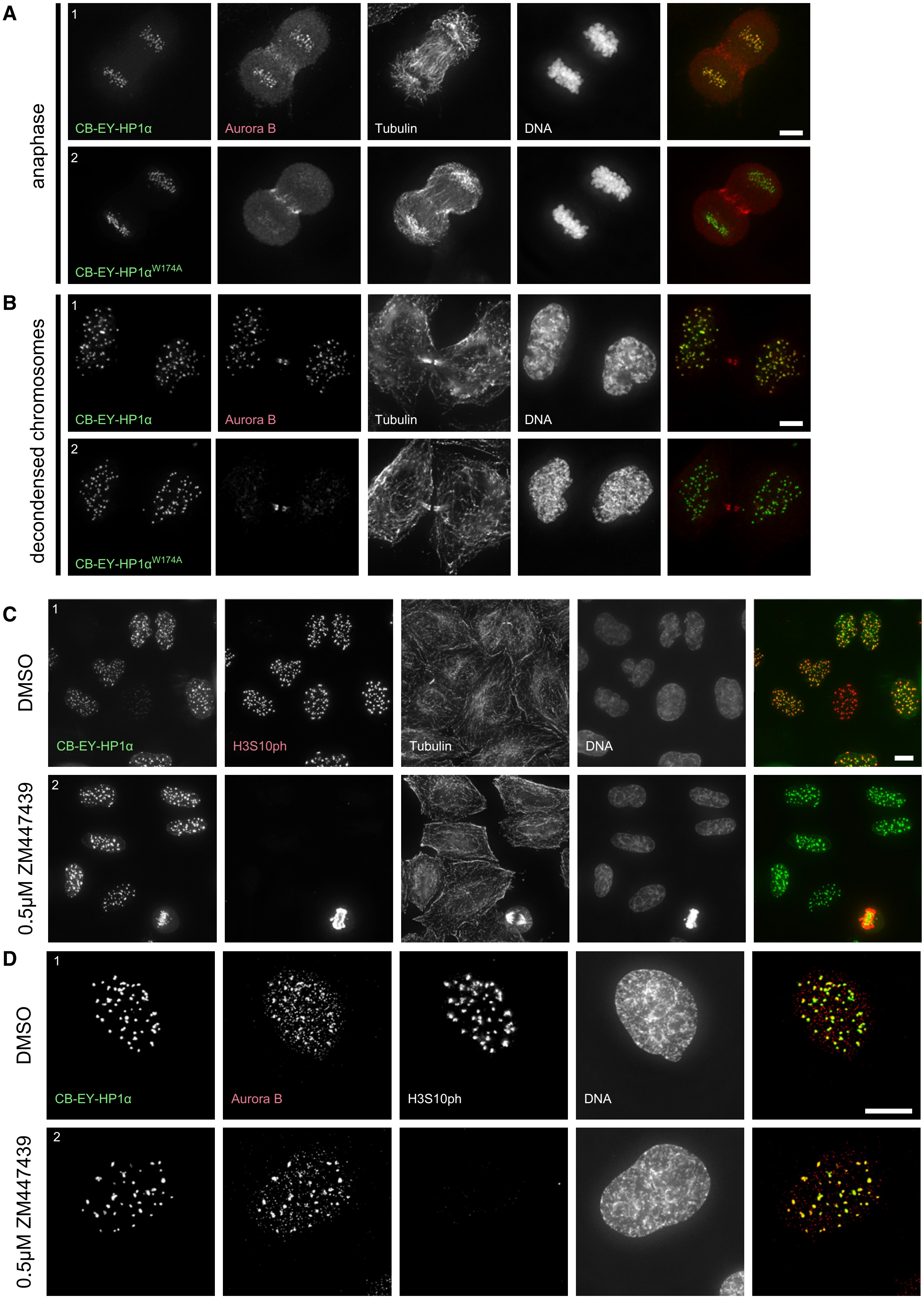

Figure EV2.

 © 2018 The Authors

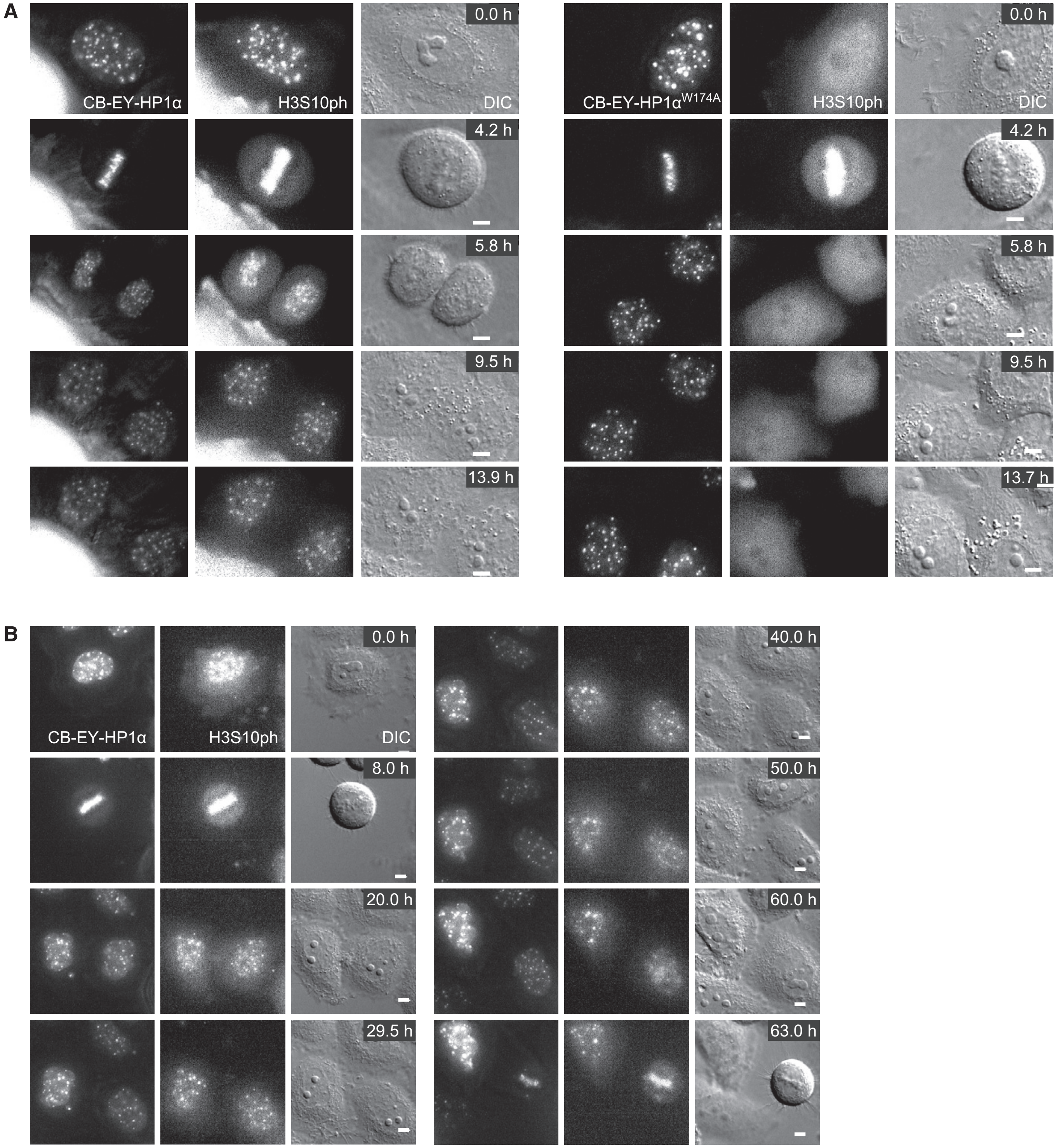

Figure EV3. Live cell imaging demonstrates that the H3S10ph signal in CB-EY-HP1α expressing cells persists throughout interphase.

A, B Stills of live cell imaging movies using CF640R-labelled Fabs against H3S10ph in HeLa cells expressing the indicated construct. Images were acquired with a 100× objective every 10 min (A) or with a 60× objective every 30 min (B) with five *z* sections every 2 μm. Scale bars, 5 μm.

Figure EV4. Live cell imaging demonstrates the robustness of the H3S10ph foci in synchronised G_2 cells that are sensitive to low concentrations of ZM447439.

A HeLa CDK1-as cells were transfected with EY-HP1 and treated 24 h after transfection with either 10 μM 1NM-PP1 (+1NM-PP1) or DMSO (−1NM-PP1) for 20 h. Cells were treated with 0.5 μM ZM447439 for 60 min before fixation, stained with Hoechst 33342 and immunostained for Aurora B and H3S10ph. Scale bar, 5 μm.

B Stills of a live cell imaging movie using Cy5-labelled Fabs against H3S10ph in HeLa CDK1-as cells treated with 10 μM 1NM-PP1. Images were acquired every 6 min with five *z* sections every 1 μm. Scale bars, 5 μm.

 © 2018 The Authors

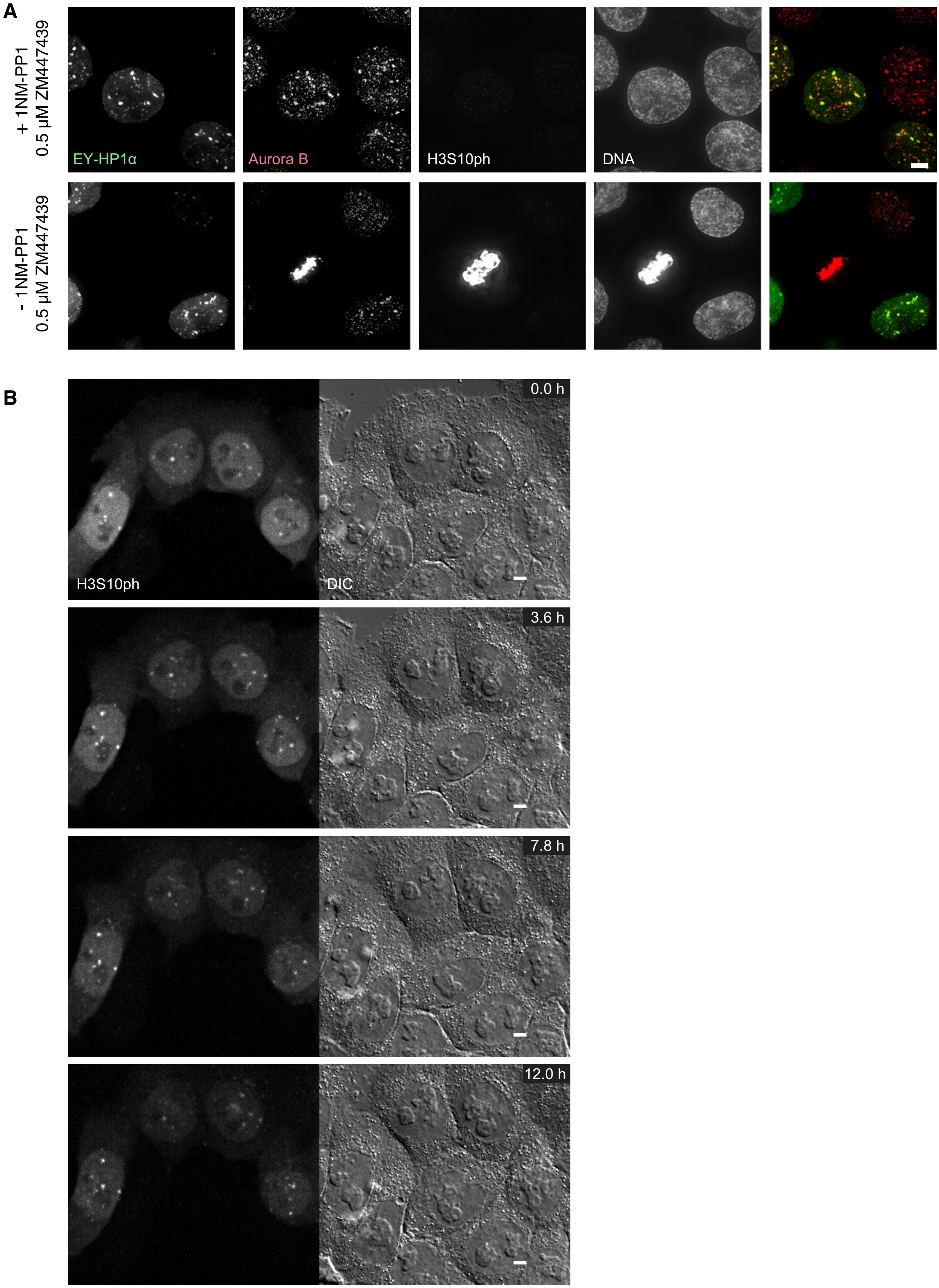
A
+ 1NM-PP1
0.5 µM ZM447439
- 1NM-PP1
0.5 µM ZM447439
EY-HP1α
Aurora B
H3S10ph
DNA
B
H3S10ph
DIC
0.0 h
3.6 h
7.8 h
12.0 h

Figure EV4.

Figure EV5. Time difference between the robust appearance of H3S10ph and H3T3ph seems to be smaller in HP1α + HP1γ double KO cells.

A Immunoblot of the indicated HP1 KO cell lines using anti-HP1α, HP1β or HP1γ antibodies and GAPDH was used as a loading control.
B Stills of live cell imaging movies using Cy5-labelled Fabs against H3S10ph in HeLa wild-type or HP1α and HP1γ double KO cells. Brightness of the far red channel was adjusted individually (0.65% difference), to account for slightly higher amount of loaded Fab fragments in the wild-type cell. Images were acquired every 6 min with five *z* sections every 1.2 μm. Scale bars, 5 μm.
C HeLa wild-type (1), or HP1α and HP1γ double KO (2) cells were stained with Hoechst 33342 and immunostained for H3S10ph and H3T3ph. Scale bar, 5 μm.
D Stills of live cell imaging movies using Alexa488-labelled Fabs against H3S10ph and CF640R-labelled Fabs against H3T3ph in HeLa wild-type or HP1α and HP1γ double KO cells. Brightness of the far red channel was adjusted individually (0.76% difference), to account for slightly higher amount of loaded Fab fragments in the HP1α and HP1γ double KO cell. Images were acquired every 10 min with five *z* sections every 1.2 μm. Scale bars, 5 μm.

© 2018 The Authors

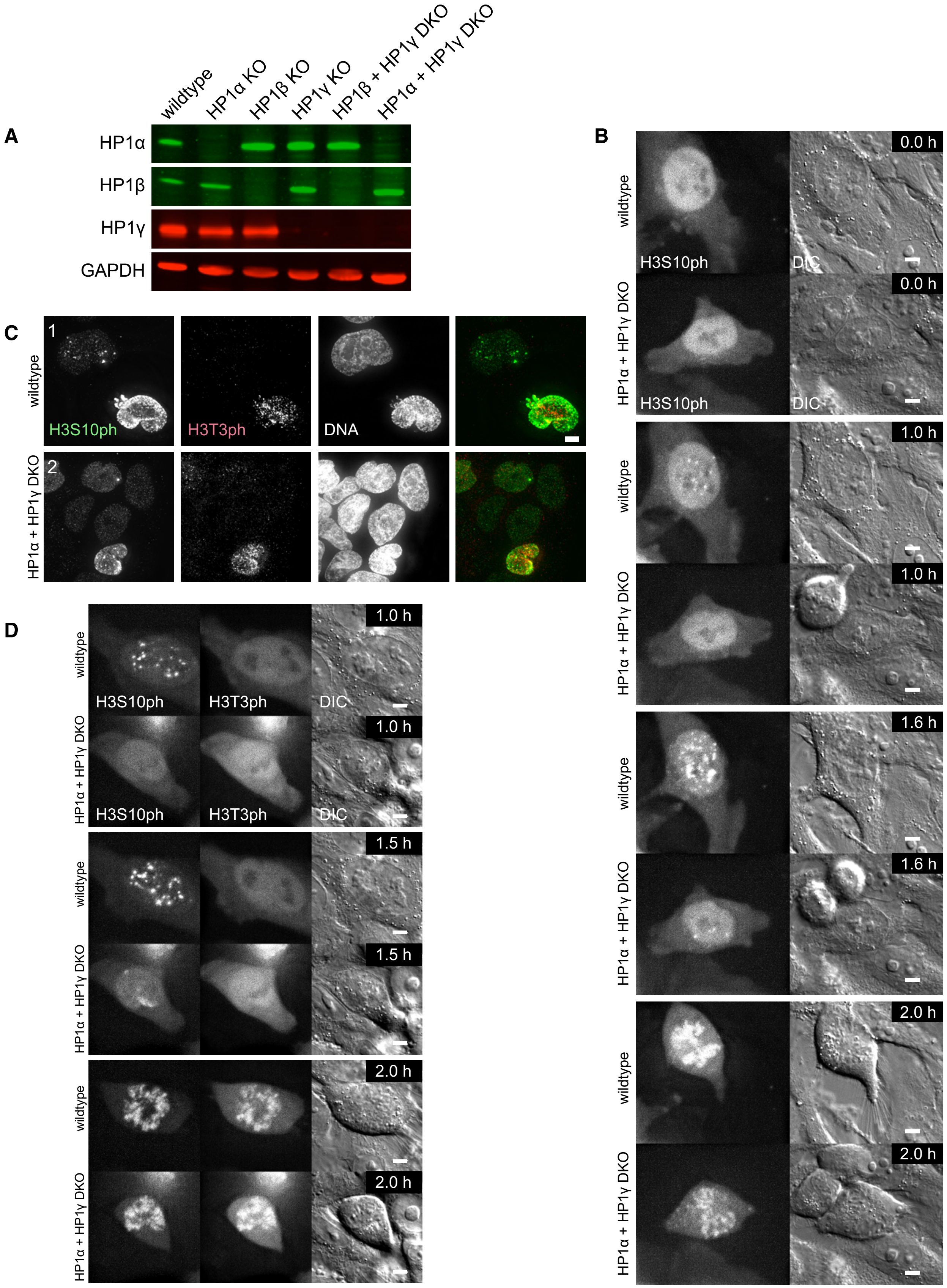
A
wildtype
HP1α KO
HP1β KO
HP1γ KO
HP1β + HP1γ DKO
HP1α + HP1γ DKO
HP1α
HP1β
HP1γ
GAPDH
B
wildtype
HP1α + HP1γ DKO
H3S10ph
DIC
0.0 h
1.0 h
1.6 h
2.0 h
C
1
2
H3S10ph
H3T3ph
DNA
D
H3S10ph
H3T3ph
DIC
1.0 h
1.5 h
2.0 h

Figure EV5.
