# Supplementary material for: HP1α targets the chromosomal passenger complex for activation at heterochromatin before mitotic entry
Source: EMBO J. 2018 Feb 21;37(6):e97677. doi: 10.15252/embj.201797677 (PMC5852645; doi:10.15252/embj.201797677)
Supplement: Supplementary file 2 — Table EV1 [file EMBJ-37-e97677-s002.docx]

| **Cell type** | **Construct** | **Expression** | **n cells: Timing** | **n cells: Defects** |
| --- | --- | --- | --- | --- |
| HeLa | CB-EY-HP1α | low | 23 | 21 |
| HeLa | CB-EY-HP1α | medium | 17 | 15 |
| HeLa | CB-EY-HP1α | high | 20 | 14 |
| HeLa | CB-EY-HP1αW174A | low | 20 | 18 |
| HeLa | CB-EY-HP1αW174A | medium | 23 | 23 |
| HeLa | CB-EY-HP1αW174A | high | 16 | 16 |
| HeLa | untransfected | - | 50 | - |
| U2OS | CB-EY-HP1α | low | 34 | 29 |
| U2OS | CB-EY-HP1α | medium | 38 | 29 |
| U2OS | CB-EY-HP1α | high | 29 | 24 |
| U2OS | CB-EY-HP1αW174A | low | 11 | 11 |
| U2OS | CB-EY-HP1αW174A | medium | 23 | 22 |
| U2OS | CB-EY-HP1αW174A | high | 14 | 12 |
| U2OS | untransfected | - | 24 | - |

Expanded View Table EV1

Numbers of cells used to determine mitotic timing (Figure 2B) and mitotic defects (Figure 2C, D) from live cell movies. Each cell line is shown either untransfected or expressing the wildtype HP1α or mutated HP1α^W174A^ tethering construct, grouped by the expression level.
