# Supplementary material for: HP1α targets the chromosomal passenger complex for activation at heterochromatin before mitotic entry
Source: EMBO J. 2018 Feb 21;37(6):e97677. doi: 10.15252/embj.201797677 (PMC5852645; doi:10.15252/embj.201797677)
Supplement: Supplementary file 3 — Movie EV1 [file EMBJ-37-e97677-s003.zip › Movie_EV1.docx]

Movie EV1: Interphase H3S10ph signal persists only in CB-EY-HP1α expressing cells.

Live cell imaging movies using CF640R-labelled Fabs against H3S10ph in HeLa cells expressing the indicated construct. Images were acquired every 10 min with 5 z sections every 2 µm. Scale bar, 5 µm.
