# Supplementary material for: HP1α targets the chromosomal passenger complex for activation at heterochromatin before mitotic entry
Source: EMBO J. 2018 Feb 21;37(6):e97677. doi: 10.15252/embj.201797677 (PMC5852645; doi:10.15252/embj.201797677)
Supplement: Supplementary file 5 — Movie EV3 [file EMBJ-37-e97677-s005.zip › Movie_EV3.docx]

Movie EV3: H3S10ph foci are highly stable in synchronised G_2_ cells.

Live cell imaging movie using Cy5-labelled Fabs against H3S10ph in HeLa CDK1-as cells treated with 10 µM 1NM-PP1. Images were acquired every 6 min with 5 z sections every 1 µm. Scale bar, 5 µm.
