# Supplementary material for: HP1α targets the chromosomal passenger complex for activation at heterochromatin before mitotic entry
Source: EMBO J. 2018 Feb 21;37(6):e97677. doi: 10.15252/embj.201797677 (PMC5852645; doi:10.15252/embj.201797677)
Supplement: Supplementary file 6 — Movie EV4 [file EMBJ-37-e97677-s006.zip › Movie_EV4.docx]

Movie EV4: H3S10 phosphorylation precedes H3T3 phosphorylation in G_2_ cells.

Live cell imaging movie using Alexa488-labelled Fabs against H3S10ph and CF640R-labelled Fabs against H3T3ph in HeLa cells. Images were acquired every 10 min with 5 z sections every 1.2 µm. Scale bar, 5 µm.
