# Supplementary material for: HP1α targets the chromosomal passenger complex for activation at heterochromatin before mitotic entry
Source: EMBO J. 2018 Feb 21;37(6):e97677. doi: 10.15252/embj.201797677 (PMC5852645; doi:10.15252/embj.201797677)
Supplement: Supplementary file 7 — Movie EV5 [file EMBJ-37-e97677-s007.zip › Movie_EV5.docx]

Movie EV5: H3S10ph foci appear only shortly before mitosis in HP1α and HP1γ double KO cells.

Live cell imaging movies using Cy5-labelled Fabs against H3S10ph in in HeLa wildtype (upper row) or HP1α + HP1γ double KO (lower row) cells. Brightness of the far red channel was adjusted individually (0.65 % difference), to account for slightly higher amount of loaded Fab fragments in the wildtype cell. Images were acquired every 6 min with 5 z sections every 1.2 µm. Scale bar, 5 µm.
