# Supplementary material for: HP1α targets the chromosomal passenger complex for activation at heterochromatin before mitotic entry
Source: EMBO J. 2018 Feb 21;37(6):e97677. doi: 10.15252/embj.201797677 (PMC5852645; doi:10.15252/embj.201797677)
Supplement: Supplementary file 8 — Movie EV6 [file EMBJ-37-e97677-s008.zip › Movie_EV6.docx]

Movie EV6: Imaging of H3T3ph and H3S10ph reveals that the time difference between the robust appearance of these marks is smaller in HP1α and HP1γ double KO cells.

Live cell imaging movies using Alexa488-labelled Fabs against H3S10ph and CF640R-labelled Fabs against H3T3ph in HeLa wildtype (upper row) or HP1α + HP1γ DKO (lower row) cells. Brightness of the far red channel was adjusted individually (0.76 % difference), to account for slightly higher amount of loaded Fab fragments in the HP1α and HP1γ double KO cell. Images were acquired every 10 min with 5 z sections every 1.2 µm. Scale bar, 5 µm.
